# Circulating long non-coding RNAs detection after heart transplantation and its accuracy in the diagnosis of acute cardiac rejection

**DOI:** 10.1186/s40364-024-00590-0

**Published:** 2024-05-12

**Authors:** Lorena Pérez-Carrillo, Isaac Giménez-Escamilla, Irene González-Torrent, Marta Delgado-Arija, Ignacio Sánchez-Lázaro, María García-Manzanares, Luis Martínez-Dolz, Manuel Portolés, Estefanía Tarazón, Esther Roselló-Lletí

**Affiliations:** 1 Clinical and Translational Research in Cardiology Unit, Health Research Institute Hospital La Fe (IISLaFe), Valencia, Spain; 2 CIBERCV, Madrid, Spain; 3 Heart Failure and Transplantation Unit, Cardiology Department, University and Polytechnic La Fe Hospital, Valencia, Spain; 4 Department of Animal Medicine and Surgery, Veterinary Faculty, CEU Cardenal Herrera University, Valencia, Spain

**Keywords:** Acute cellular rejection, Biomarkers, Diagnostic, lncRNAs, Transplantation

## Abstract

Long non-coding RNAs (lncRNAs) are closely implicated in biological processes and diseases with high inflammatory components. These molecules exhibit significant temporal and tissue specificity. However, the expression and function of lncRNAs have not been studied in patients after heart transplantation. Thus, we aimed to identify circulating lncRNAs in these patients and evaluate their diagnostic capacity as potential biomarkers for the non-invasive detection of acute cellular rejection (ACR). For them, we performed a transcriptomic study based on ncRNA-seq technology to detect lncRNAs in serum samples, matched to routine endomyocardial biopsies, from patients without rejection episode (0R, *n* = 12) and with mild (1R, *n* = 16) or moderate-severe (≥ 2R, *n* = 12) ACR. We identified 11,062 circulating lncRNAs in the serum of patients after heart transplantation. Moreover, 6 lncRNAs showed statistically significant expression when the different ACR grades were compared. Among them, AC008105.3, AC006525.1, AC011455.8, AL359220.1, and AC025279.1 had relevant diagnostic capacity for detection of ≥ 2R (AUC of 0.850 to 1.000) and 1R (AUC of 0.750 to 0.854) grades, along with high specificity and positive predictive values (≥ 83%). In addition, AL359220.1 and AC025279.1 were independent predictors for the presence of moderate-severe ACR (odds ratio = 31.132, *p* < 0.01 and C statistic = 0.939, *p* < 0.0001; odds ratio = 18.693, *p* < 0.05 and C statistic = 0.902, *p* < 0.001; respectively). In conclusion, we describe, for the first time, circulating lncRNAs after heart transplantation as potential candidates for non-invasive detection of ACR. AL359220.1 and AC025279.1 showed excellent diagnostic capability correlating with the severity episode and were strong independent predictors of rejection.

**Supplementary Information:**

The online version contains supplementary material available at 10.1186/s40364-024-00590-0.

## To the editor

Heart transplantation remains the definitive treatment for patients with advanced heart failure or congenital heart disease [1]. Cardiac allograft transplantation is associated with a significant risk of rejection [2], and one of the main events is acute cellular rejection (ACR) [3]. The endomyocardial biopsy (EMB) screening is the gold standard technique for diagnosing cardiac rejection despite its technical limitations and risk to patients [4]. Therefore, alternatives such as liquid biopsy are an interesting source of non-invasive information for the follow-up of heart transplant patients. In recent decades, research has focused on studying genes that code for proteins as potential circulating biomarkers for cardiac rejection [5, 6]. However, protein-coding genes represent less than 2% of the total genome, and more than 90% of the genome is transcribed into non-coding RNAs (ncRNAs). Recent investigations are beginning to include long non-coding RNAs (lncRNAs) as critical molecules in the development of cardiovascular diseases, being proposed as biomarkers and therapeutic targets [7]. In addition, animal studies have indicated alterations in several lncRNAs, which might be related to ACR episodes [8].

**Fig. 1:**
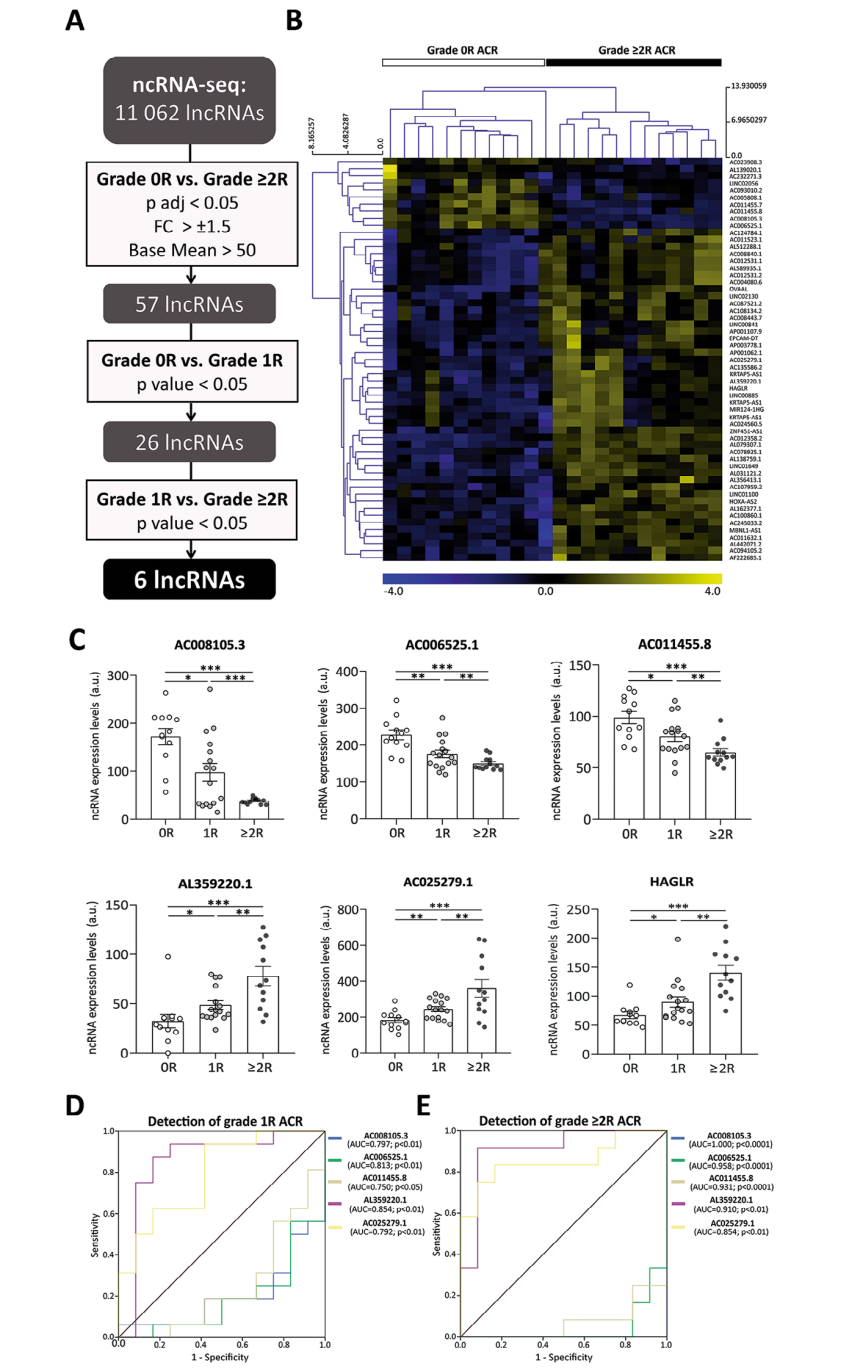
**A**. Schematic overview of identification strategy of candidate long non-coding RNAs (lncRNAs) for the detection of acute cellular rejection (ACR), both mild (1R) and moderate-severe (≥ 2R) grades. FC, fold change. P adj, p adjusted. **B.** Hierarchical clustering shows a distinguishable lncRNA expression profiling among patient serum samples, visualized in a heatmap. Colors depict the relative expression level of each molecule, with blue being the lowest and yellow the highest. **C**. Dot plot graph of circulating expression levels of lncRNAs selected as potential biomarkers for the detection of acute cellular rejection (ACR). Comparison between the non-rejection group (grade 0R) and the different grades of acute rejection of heart allografts (ACR grade 1R and grade ≥ 2R), as well as between mild (1R) and moderate-severe (≥ 2R) grades. Data are represented as the mean ± SEM. A.u., arbitrary units. *p < 0.05, **p < 0.01, ***p < 0.0001. **D.** Serum lncRNAs receiver operating characteristic curves for the detection of cardiac ACR of mild (1R) grade. AUC, area under the curve. **E**. Serum lncRNAs receiver operating characteristic curves for the detection of cardiac ACR of moderate-severe (≥ 2R) grades. AUC, area under the curve

Thus, we performed a ncRNA-sequencing study (Supplementary material) to identify the presence of lncRNAs in 40 consecutive serum samples matched to routine EMB from heart transplant patients diagnosed for ACR (grade 0R ACR, *n* = 12; grade 1R ACR, *n* = 16; and grade ≥ 2R ACR, *n* = 12) (Table S1). Specifically, 11,062 circulating lncRNAs were detected. Next, we established a series of criteria for selecting the lncRNAs with the most significant potential as possible biomarkers of ACR, obtaining 57 lncRNAs candidates (Fig. 1A). These showed two divergent lncRNA expression profiles, indicating a clear distinction between ≥ 2R and 0R grades. (Fig. 1B). Six of these lncRNAs (AC008105.3, AC006525.1, AC011455.8, AL359220.1, AC025279.1, and HAGLR) presented statistically significant expression (*p* < 0.05) when the different ACR grades were compared, and their expression levels changed proportionally to the severity of the rejection (Fig. 1C). Among them, 5 showed relevant diagnostic capacity with an AUC ≥ 0.750 (Fig. 1D) and AUC > 0.850 (Fig. 1E) for the detection of 1R and ≥ 2R grades, respectively. Additionally, we observed high specificity and positive predictive values (≥ 83%) for these circulating lncRNAs (Table S2). Furthermore, to investigate whether circulating lncRNAs are independent predictors of grade ≥ 2R ACR binary logistic regressions were performed. The best models were obtained for AL359220.1 and AC025279.1 (value of fold change ≥ 1.5, optimum cut-off point obtained from the ROC curve) adjusted for age, sex and NT-proBNP levels. Specifically, AL359220.1 showed an odds ratio of 31.132 (95% CI 2.340–414.2, *p* < 0.01) with a C statistic of 0.939 (95% CI 0.848–1.000, *p* < 0.0001), and AC025279.1 showed an odds ratio of 18.693 (95% CI 1.511–231.2, *p* < 0.05) with a C statistic of 0.902 (95% CI 0.777–1.000, *p* < 0.001).

The main function of lncRNAs is the regulation of gene expression through miRNA interactions [9]. Therefore, due to the current lack of knowledge of the specific biological functions of the described lncRNAs, we studied the possible function of lncRNAs as miRNA sponges in ACR. For this, we use our expression data set for miRNAs and lncRNAs derived from ncRNA sequencing to obtain a thermodynamic measure of the lncRNA-miRNA interaction (Supplementary material). AC006525.1 and AC011455.8 showed a correlation > l0.60 L and a favourable interaction (minimum Gibbs free energy of − 18 kcal/mol) with respect to randomness within the same RNA length for several miRNAs (Table 1). Then, a miRNA target prediction analysis was performed. Only experimentally validated miRNA targets previously published through reporter assays were included to ensure a high level of biological relevance (Table S3).

Nowadays, all commercial alternatives to EMB are restricted to reference commercial laboratories, such AlloMap [10] and percent donor-derived cell-free DNA (%ddcfDNA) [11]. Additionally, the costs of these tests are similar to that of EMB and not available in all countries [12]. For these reasons, although this is a pilot study with a small cohort and involving only a single center, our findings have provided substantial evidence and represent a necessary first step. Limiting factors could be addressed in future research, such as validation in a large multicenter cohort in which the response to the treatment will also be analyzed and the use of a simple standardized technique in all clinical laboratories for the identification of these potential lncRNAs as biomarkers of cardiac rejection.

**Table 1:** Significant lncRNA-miRNA interactions

lncRNA	miRNA	Size	kcal.mol	Predict	Fold Change
AC006525.1	miR-3605-3p	22	-21,11	-17,12715	-3,98285
	miR-3178	16	-19,06	-16,44425	-2,61575
AC011455.8	miR-3178	16	-18,32	-16,54603	-1,77397
	miR-3605-3p	22	-16,51	-13,99351	-2,51649
	miR-6787-5p	21	-18,07	-14,41893	-3,65107

AC006525.1 and AC011455.8 showed a correlation > l0.60 L and a favourable interaction (minimum Gibbs free energy of − 18 kcal/mol) with respect to randomness within the same RNA length for these miRNAs. *P* ≤ 0.10

### Electronic supplementary material

Below is the link to the electronic supplementary material.

Supplementary Material 1

## Data Availability

The datasets used and/or analysed during the current study are available from the corresponding author on reasonable request.

## Abbreviations

ACR: Acute cellular rejection
AUC: Area under the curve
EMB: Endomyocardial biopsies
FC: Fold change
ISHLT: International Society for Heart and Lung Transplantation
LncRNA: Long non-coding RNA
NT-proBNP: N-terminal pro–B-type natriuretic peptide
ROC: Receiver operating characteristic
%ddcfDNA: Percent donor-derived cell-free DNA
