# Supplementary material for: Circulating long non-coding RNAs detection after heart transplantation and its accuracy in the diagnosis of acute cardiac rejection
Source: Biomark Res. 2024 May 12;12:49. doi: 10.1186/s40364-024-00590-0 (PMC11089702; doi:10.1186/s40364-024-00590-0)
Supplement: Supplementary file 1 — Supplementary Material 1 [file 40364_2024_590_MOESM1_ESM.docx]

**Circulating long non-coding RNAs detection after heart transplantation and its accuracy in the diagnosis of acute cardiac rejection**

Lorena Pérez-Carrillo, BSc; Isaac Giménez-Escamilla, BSc; Irene González-Torrent, BSc; Ignacio Sánchez-Lázaro, PhD; María García-Manzanares, PhD; Luis Martínez-Dolz^a^, PhD; Manuel Portolés, PhD; Estefanía Tarazón, PhD; Esther Roselló-Lletí, PhD

1. **Materials and methods**
   1. **Study cohort**

This study included 40 consecutive serum samples matched to routine endomyocardial biopsy (EMB) from heart transplant patients (>18 years). Of the patients studied, 28 samples had a diagnosis of biopsy-proven allograft rejection (grade 1R acute cellular rejection (ACR), n = 16; and grade ≥2R ACR, n = 12 (grade 2R, n = 11 and grade 3R, n = 1)), these were compared with 12 samples from patients who did not experience allograft rejection (grade 0R ACR). The samples and associated clinical data were collected from follow-up visits of cardiac transplantation recipients from The University and Polytechnic Hospital La Fe (October 2016 to April 2017). At the time of EMB, blood samples were collected for laboratory analysis. The serum was separated by centrifugation at 1500 x g for 10 min at 4°C, aliquoted, and immediately stored at −80°C until analysis. Cardiac rejection episodes were assessed according to the International Society for Heart and Lung Transplantation (ISHLT) consensus report [1]. Experimenters were blind to group allocation and outcome assessment. The study was approved by the Ethics Committee (Biomedical Investigation Ethics Committee of University and Polytechnic Hospital La Fe of Valencia, Spain). It was conducted following the principles outlined in the Declaration of Helsinki [2]. Prior to sample collection, informed consent was obtained from each patient.

The characteristics of the patients at the time of biopsy and blood sampling were obtained and are presented in Table S1. The ACR and non-ACR groups were homogeneous in variables such as age (42-51 years), sex (75-94 % men), body mass index (24-25 kg/m^2^), hypertension (31-58 %), diabetes (50-63%), lymphocyte number (1.5-2.0 thousands/mm^3^), and troponin T levels (15-25 ng/L), among others. Moreover, all patients received immunosuppressive and induction therapy. Only patients with moderate-severe grades displayed significant differences in specific clinical characteristics when we compared them with the non-ACR group. The time between transplantation and study enrolment was lower in grade ≥2R ACR. We found higher values in left ventricle end-systolic diameter, mean right atrial pressure, and systolic right ventricular pressure in grade ≥2R ACR. In addition, this group showed an increase in N-terminal pro-B-type natriuretic peptide (NT-proBNP) levels.

- 1. **RNA isolation and RNA sequencing**

RNA isolation and RNA sequencing were performed as described by Tarazón et al. [3]. RNA extraction was carried out using NucleoSpin® miRNA Plasma of Macherey Nagel (Dürer, Germany), following the protocol and instructions provided by the manufacturer. RNA quantification was performed using a NanoDrop 1000 spectrophotometer and the Qubit 3.0 fluorometer (Thermo Fisher Scientific, MA, USA). The purity and integrity of RNA samples were determined using the RNA 6000 Nano Kit and Small RNA Kit with 0.8% agarose gel and the Agilent 2100 Bioanalyzer (Agilent Technologies, CA, USA). Extracted RNA were only considered valid samples with a 260/280 absorbance ratio greater than 2.0, and an RNA integrity number (RIN software algorithm), equal to or greater than 7.

cDNA libraries were obtained following Illumina’s recommendations. Briefly, 3´ and 5´ adapters were sequentially ligated to RNA prior to reverse transcription and cDNA synthesis. Size selection was performed using a 6% polyacrylamide gel. The quality and quantity of cDNA libraries were analyzed using the High-Sensitivity D1000 ScreenTape Assay and the 4200 TapeStation System (Agilent Technologies, CA, USA). cDNA libraries were then pooled and sequenced by two lanes of 100 bp paired-end sequencing using an Illumina HiSeq 2500 sequencer.

Quality control of the raw sequence data was performed using FastQC software. The raw paired‐end reads were mapped against the human hg38 genome using the bowtie algorithm [4]. Insufficient quality reads, with a phred score ≤ 20, were eliminated using the SAMtools method [5]. RNA quantification was then estimated using HTSeq software (version 0.6.0) [6].

- 1. **lncRNA-miRNA interaction**

The correlation between lncRNAs and miRNAs was calculated using Spearman's value with the normalised counts obtained from Deseq2 [7]. The threshold of +/- 0.50 was used for a significant correlation between RNAs interactions. For the energetic interaction, the IntaRNA method was applied [8]. For the statistically favourable interaction, random sampling was used for thresholding and assessment of statistical significance. All values were normalised by the length of the lntaRNAs. The p-value was calculated and 0.10 was selected as the threshold. Possible lcRNAs-miRNAs interactions were considered as possible interactions if the correlation expression and energy interaction were significant.

- 1. **Statistical analysis**

Clinical characteristics were expressed as mean ± standard deviation for continuous variables and percentages for discrete variables. Results for each variable were assessed for normality using the Kolmogorov–Smirnov test. Continuous variables not following a normal distribution were compared using the Mann-Whitney test, and variables with a normal distribution were compared using the Student’s t-test. Fisher’s exact test was used to compare discrete variables. For RNA sequencing analysis, differential expression analyses between conditions were assessed using the DESeq2 method (version 3.4) [7]. We used the false discovery rate (FDR) method for adjusts the original p value using the number of tests. Differentially expressed RNAs with fold change (FC) values ± 1.5, and with FDR adjusted p ≤ 0.05 were included to avoid the identification of false positives across the differential expression data [7]. The diagnostic capability of serum markers for the presence of transplant rejection was assessed by the construction of receiver operating characteristic (ROC) curves. p < 0.05 was considered statistically significant. To determine whether lncRNAs were an independent predictor for grade ≥2R ACR, binary logistic regression analyses were performed with all relevant variables included in the models. An incremental approach was used for the continuous variables (the calculated odds ratios are related to the increase in the indicated continuous variable by a given amount), and dichotomous risk factors were coded with an indicator variable of 1 for having the condition and 0 for its absence. Discrimination was assessed through the C statistic. All statistical analyses were performed using R statistic and SPSS software (version 20.0; SPSS Inc., IL, USA).

1. **Discussion**

In recent years, many lncRNAs have been identified along with their role in different biological functions. In addition, a close relationship between lncRNAs expression and the development of diseases with an inflammatory component, including allograft rejection, have been described [9]. However, the function of lncRNAs in human cardiac rejection has yet to be explored. In this study, we detected, for the first time, circulating lncRNAs in the serum of patients after heart transplantation. We highlight the potential diagnostic capacity of five lncRNAs (AC008105.3, AC006525.1, AC011455.8, AL359220.1, and AC025279.1) capable of identifying the different types of ~~acute cellular rejection~~ ACR with high specificity. In addition, AL359220.1 and AC025279.1 were independent predictors of grade ≥2R ACR, after adjusting the model for age, sex, and NT-proBNP levels.

EMB is the gold standard technique for the diagnosis of acute rejection, which consists of a manual histological examination of tissue stained with hematoxylin and eosin for the identification of infiltration by cells of the immune system. This technique still has limited inter-observer and intra-observer reproducibility [10]. Thus, several alternatives have been proposed, such as the development of artificial intelligence systems [11] or the detection of gene expression profiling in EMB [12]. However, all these techniques are invasive and associated with both procedural and severe long-term sequelae risks when performed repeatedly [13]. In this context, alternatives such as liquid biopsy are an interesting source of non-invasive information for the follow-up of heart transplant patients. The first included in ISHLT guidelines for the non-invasive detection of ~~acute cellular rejection~~ (ACR) is a gene expression profiling AlloMap. Nevertheless, this method lacks excellent positive predictive value and can only be used to rule out the presence of ACR grade 2R or more significant [14]. Detection of grade 1R rejection is essential for an adequate response, since it avoids unnecessary exposure of patients to the adverse effects of immunosuppression for levels of rejection that can resolve spontaneously. Furthermore, it has been shown that approximately 15% progressed to more severe degrees of rejection, especially in those cases detected during early post-transplantation [15]. Other genomic analyses have been emerging, such as percent donor-derived cell-free DNA (%ddcfDNA) [16–18]. However, they still present some limitations such as the inability to discriminate between the different rejection grades, so the replacement of EMB remains a challenge. Nowadays, all commercial alternatives to EMB ~~endomyocardial biopsy~~ are restricted to reference commercial laboratories, requiring specimens to be sent, and delays in result release. Additionally, the costs of these tests are similar to that of EMB and not available in all countries [19]. For these reasons, studies focused and targeted to the design of specific quantitative polymerase chain reaction (qPCR) based on standard and inexpensive reagents and amenable to widespread local implementation are needed. In this sense, our findings have provided substantial evidence and represent a necessary first step.

On the other hand, to reduce the incidence of rejection, patients receive individualised immunosuppressive regimens after transplantation. Despite medication, acute rejection remains one of the most common complications in post-transplant patients [20]. Thus, the identification of new therapeutic targets is also necessary. The cell type-specific expression and dysregulated transcript levels of lncRNAs in diseased conditions make them suitable biomarker candidates [21]. In addition, implications in the immune system, particularly in allotransplant rejection, have been described [22–24]. Therefore, lncRNAs could be considered potential targets for prediction, prognosis, diagnosis, and treatment of cardiac rejection.

lncRNAs constitute the vast majority of the non-protein coding transcriptome [25]. Only a few transcripts have been described in relation to structure, function, and impact on cellular processes or cardiovascular disease development [21]. Among the five described lncRNAs accurately detectable in the serum of patients after heart transplantation, only the specific function of AC008105.3 has been described. AC008105.3, also known as FMNL1 Divergent Transcript (FMNL1-DT), FMNL1 is a molecule expressed in cells derived from hematopoietic lineage. It is involved in the reorientation of the microtubule organizing center to the immunological synapse and T-cell cytotoxicity [26]. Sun et al.[27] identified AC008105.3 as one of the key molecules in the lncRNA signature related to the immune system with the ability to predict the prognosis for kidney renal clear cell carcinoma. In addition, RNAs can present post-translational modifications, such as specific methylations, related to the development of diseases. Specifically, N6-methyladenosine (m6A)-related lncRNAs play crucial roles in tumor prognosis. Zhao et al.[28] described AL359220.1 (or PRKCH-AS1) as an m6A-related lncRNA, involved in the clinical prognosis of lung adenocarcinoma. However, specifics functions and interactions in the lncRNAs AC006525.1, AC011455.8, and AC025279.1 have not been described so far.

A close relationship has been described between lncRNAs and miRNAs, acting as modulators of the transcriptome. lncRNAs-miRNAs interactions are quickly emerging as essential mechanisms underlying the functions of non-coding RNAs [29]. These interactions lead to miRNA-triggered RNA decay, competition between miRNA and lncRNA for the same mRNA target, and lncRNAs acting as sponges for miRNAs, preventing interaction with their target [30]. Yet, some of these interactions still need to be discovered, and they come from studies under controlled conditions, ignoring the efficiency of interactions in specific pathological conditions [31]. We described in patients after heart transplant strong interactions between the lncRNAs AC006525.1 and AC011455.8, and several miRNAs. Both lncRNAs were correlated with miR-3178 and miR-3605-3p, requiring future studies to elucidate the mechanisms involved in the development of this pathology. miR-3178 presented multiples validated targets [32–35]. Specifically, miR-3178 was described as a direct target of *RHOB* in pancreatic cancer [36]. Rhob belongs to the family of small GTPases, and it is involved in the control of endocytic/ vesicular trafficking and has wide-ranging functions in the membrane trafficking pathway [37]. Moreover, Skelton et al. [38] described the relevant role of Rhob in developing cardiac rejection. This molecule is involved in antigen presentation by dendritic cells through Endoplasmic Reticulum/Golgi/vesicular trafficking pathways regulation. In addition, studies in animal models showed a key role of lncRNAs in the induction of antigenic tolerance of dendritic cells after transplantation [39,40]. In addition, lncRNA AC011455.8 showed a strong interaction with miR-6787-5p. Tubita et al. [41] described an upregulation of this miRNA in colorectal cancer associated with the underexpression of molecules related to chromatin organisation, DNA packaging, and cell cycle, which could be an epigenetic response mechanism following rapamycin treatment after kidney transplantation.

Our study has some limitations and the results must be interpreted within this context. This investigation is focused on ~~acute cellular rejection~~ ACR and has not specifically evaluated antibody-mediated rejection. In addition, our study only involved a single center. However, we believe that our findings have provided substantial evidence and represent a necessary first step to support future research in which these limiting factors could be addressed, such as validation in a large patient cohort.

In conclusion, we describe, for the first time, circulating lncRNAs after heart transplantation as potential candidates for non-invasive detection of ACR ~~acute cellular rejection~~. AL359220.1 and AC025279.1 showed excellent diagnostic capability correlating with the severity episode and were strong independent predictors of rejection.

**Table S1.** Patient characteristics at the time of biopsy and blood sample extraction.

|  | Non-ACR  (n=12) | ACR  (n=28) | |
| --- | --- | --- | --- |
|  |  | 1R ACR  (n=16) | ≥2R ACR  (n=12) |
| Age, years | 48 ± 15 | 51 ± 10 | 42 ± 15 |
| Male sex (%) | 75 | 94 | 75 |
| Indication for cardiac transplantation |  |  |  |
| Ischemic cardiomyopathy (%) | 25 | 50 | 25 |
| Idiopathic dilated cardiomyopathy (%) | 42 | 31 | 58 |
| Other^#^ (%) | 33 | 19 | 17 |
| Time between transplantation and study enrolment, months | 8.1 ± 3.8 | 6.4 ± 3.2 | 3.8 ± 4.2* |
| Body mass index (kg/m^2^) | 25 ± 5 | 25 ± 3 | 24 ± 3 |
| Hypertension (%) | 58 | 31 | 42 |
| Diabetes mellitus (%) | 58 | 63 | 50 |
| Dyslipemia (%) | 42 | 56 | 25 |
| Echo-Doppler study |  |  |  |
| Ejection fraction (%) | 72 ± 8 | 64 ± 9 | 70 ± 10 |
| LV end systolic diameter (mm) | 25 ± 3 | 28 ± 4 | 31 ± 3* |
| LV end diastolic diameter (mm) | 41 ± 3 | 44 ± 5 | 45 ± 4 |
| Hemodynamic parameters |  |  |  |
| Mean right atrial pressure (mm Hg) | 3.6 ± 1.8 | 5.9 ± 3.2 | 7.7 ± 1.5* |
| Systolic right ventricular pressure (mm Hg) | 33 ± 5 | 36 ± 7 | 42 ± 4* |
| Diastolic right ventricular pressure (mm Hg) | 4.2 ± 1.6 | 5.9 ± 3.3 | 8.7 ± 3.8 |
| Immunosuppressive therapy |  |  |  |
| Tacrolimus (%) | 100 | 100 | 100 |
| Mycophenolic acid (%) | 100 | 95 | 100 |
| Steroids (%) | 100 | 95 | 100 |
| Induction therapy  Basiliximab (%) | 100 | 100 | 100 |
| Neutrophils (thousands/mm^3^) | 4.8 ± 4.1 | 3.7 ± 1.7 | 8.2 ± 6.3 |
| Leukocytes (thousands/mm^3^) | 7.1 ± 3.8 | 6.2 ± 2.1 | 11.0 ± 6.3 |
| Lymphocytes (thousands/mm^3^) | 1.5 ± 0.53 | 1.8 ± 0.6 | 2.0 ± 0.8 |
| Hemoglobin (mg/dL) | 11.6 ± 2.4 | 12.6 ± 1.4 | 11.9 ± 1.7 |
| Hematocrit (%) | 37 ± 8 | 40 ± 4 | 37 ± 4 |
| NT-proBNP (pg/mL) | 152 (113-467) | 280 (122-572) | 1209 (736-2382)* |
| Troponin T (ng/L) | 19 (11-66) | 15 (10-21) | 25 (13-40) |

LV, left ventricular; NT-proBNP, N-terminal fragment of B-type natriuretic peptide. ^#^ myocarditis and arrhythmogenic cardiomyopathy. *p<0.05 between non-ACR and ≥2R ACR.

**Table S2.** Receiver-operating characteristic curve of circulating lncRNAs for detecting acute cellular rejection.

| **lncRNA** |  | **AUC** | ***P* value** | **95% CI** | **SS** | **SP** | **PPV** | **NPV** |
| --- | --- | --- | --- | --- | --- | --- | --- | --- |
| AC008105.3 | **1R** | 0.797 | p < 0.01 | 0.628-0.966 | 63 | 83 | 83 | 63 |
|  | **≥2R** | 1.000 | p < 0.0001 | 1.000-1.000 | 100 | 83 | 86 | 100 |
| AC006525.1 | **1R** | 0.813 | p < 0.01 | 0.652-0.973 | 31 | 100 | 100 | 52 |
|  | **≥2R** | 0.958 | p < 0.0001 | 0.887-1.000 | 58 | 100 | 100 | 71 |
| AC011455.8 | **1R** | 0.750 | p < 0.05 | 0.563-0.973 | 13 | 100 | 100 | 43 |
|  | **≥2R** | 0.931 | p < 0.0001 | 0.830-1.000 | 67 | 100 | 100 | 75 |
| AL359220.1 | **1R** | 0.854 | p < 0.01 | 0.682-1.000 | 31 | 92 | 83 | 50 |
|  | **≥2R** | 0.910 | p < 0.01 | 0.782-1.000 | 75 | 92 | 90 | 79 |
| AC025279.1 | **1R** | 0.792 | p < 0.01 | 0.622-0.961 | 31 | 92 | 86 | 52 |
|  | **≥2R** | 0.854 | p < 0.01 | 0.691-1.000 | 58 | 92 | 88 | 69 |

Sensitivities, specificities, and predictive values (%) for the diagnostic of acute cellular rejection (cut-off point fold change ≥ ± 1.5). AUC, area under the curve; CI, confidence interval; NPV, negative predictive value; PPV, predictive positive value; SP, specificity; SS, sensitivity.

**Table S3.** Experimentally validated miR3178 target genes.

| **miRNA** | **Target** | **Function** | **References** |
| --- | --- | --- | --- |
| miR-3178 | *RHOB* | Angiogenesis, apoptosis, cell adhesion, differentiation, protein transport | [36] |
|  | *HTRIA* | Regulation of behavior | [32] |
|  | *MYOD1* | Differentiation, myogenesis, transcription regulation | [33] |
|  | *TRIOBP* | Cell cycle, cell division, mitosis | [34] |
|  | *NOTCH1* | Angiogenesis, differentiation, Notch signaling pathway, transcription regulation | [35] |

**References**

1. Stewart S, Winters GL, Fishbein MC, Tazelaar HD, Kobashigawa J, Abrams J, et al. Revision of the 1990 Working Formulation for the Standardization of Nomenclature in the Diagnosis of Heart Rejection. J Heart Lung Transplant. 2005;24:1710–20.

2. Macrae DJ. The Council for International Organizations and Medical Sciences (CIOMS) Guidelines on Ethics of Clinical Trials. Proc Am Thorac Soc. 2007;4:176–9.

3. Tarazón E, Pérez-Carrillo L, García-Bolufer P, Triviño JC, Feijóo-Bandín S, Lago F, et al. Circulating mitochondrial genes detect acute cardiac allograft rejection: Role of the mitochondrial calcium uniporter complex. Am J Transplant. 2021;21:2056–66.

4. Langmead B, Trapnell C, Pop M, Salzberg SL. Ultrafast and memory-efficient alignment of short DNA sequences to the human genome. Genome Biol. 2009;10:R25.

5. Li H, Handsaker B, Wysoker A, Fennell T, Ruan J, Homer N, et al. The Sequence Alignment/Map format and SAMtools. Bioinformatics. 2009;25:2078–9.

6. Anders S, Pyl PT, Huber W. HTSeq—a Python framework to work with high-throughput sequencing data. Bioinformatics. 2015;31:166–9.

7. Love MI, Huber W, Anders S. Moderated estimation of fold change and dispersion for RNA-seq data with DESeq2. Genome Biol. 2014;15:550.

8. Mann M, Wright PR, Backofen R. IntaRNA 2.0: enhanced and customizable prediction of RNA–RNA interactions. Nucleic Acids Res. 2017;45:W435–9.

9. Chen J, Ao L, Yang J. Long non-coding RNAs in diseases related to inflammation and immunity. Ann Transl Med. 2019;7:494–494.

10. Kobashigawa JA. The Search for a Gold Standard to Detect Rejection in Heart Transplant Patients. Circulation. 2017;135:936–8.

11. Lipkova J, Chen TY, Lu MY, Chen RJ, Shady M, Williams M, et al. Deep learning-enabled assessment of cardiac allograft rejection from endomyocardial biopsies. Nat Med. 2022;28:575–82.

12. Bodez D, Hocini H, Tchitchek N, Tisserand P, Benhaiem N, Barau C, et al. Myocardial Gene Expression Profiling to Predict and Identify Cardiac Allograft Acute Cellular Rejection: The GET-Study. PLoS One. 2016;11:e0167213.

13. From AM, Maleszewski JJ, Rihal CS. Current Status of Endomyocardial Biopsy. Mayo Clin Proc. 2011;86:1095–102.

14. Crespo-Leiro MG, Stypmann J, Schulz U, Zuckermann A, Mohacsi P, Bara C, et al. Clinical usefulness of gene-expression profile to rule out acute rejection after heart transplantation: CARGO II. Eur Heart J. 2016;37:2591–601.

15. Winters GL, Loh E, Schoen FJ. Natural History of Focal Moderate Cardiac Allograft Rejection. Circulation. 1995;91:1975–80.

16. Agbor-Enoh S, Shah P, Tunc I, Hsu S, Russell S, Feller E, et al. Cell-Free DNA to Detect Heart Allograft Acute Rejection. Circulation. 2021;143:1184–97.

17. Velleca A, Shullo MA, Dhital K, Azeka E, Colvin M, DePasquale E, et al. The International Society for Heart and Lung Transplantation (ISHLT) guidelines for the care of heart transplant recipients. J Heart Lung Transplant. 2023;42:e1–141.

18. Di W, Ran Q, Yang H, Lu J, Hou Y, Wang X, et al. Use of graft‐derived cell‐free DNA as a novel biomarker to predict allograft function after kidney transplantation. Int J Urol. 2021;28:1019–25.

19. Baran DA. Anything But a Biopsy: The Quest for Noninvasive Alternatives in Heart Transplantation. Transplantation. 2023;107:1875–6.

20. Lund LH, Khush KK, Cherikh WS, Goldfarb S, Kucheryavaya AY, Levvey BJ, et al. The Registry of the International Society for Heart and Lung Transplantation: Thirty-fourth Adult Heart Transplantation Report—2017; Focus Theme: Allograft ischemic time. The Journal of Heart and Lung Transplantation. 2017;36:1037–46.

21. Viereck J, Thum T. Circulating Noncoding RNAs as Biomarkers of Cardiovascular Disease and Injury. Circ Res. 2017;120:381–99.

22. Zhang Z, Tang Y, Zhuang H, Lin E, Xie L, Feng X, et al. Identifying 4 Novel lncRNAs as Potential Biomarkers for Acute Rejection and Graft Loss of Renal Allograft. J Immunol Res. 2020;2020:1–22.

23. Qiu J, Chen Y, Huang G, Zhang Z, Chen L, Na N. Transforming growth factor-β activated long non-coding RNA ATB plays an important role in acute rejection of renal allografts and may impacts the postoperative pharmaceutical immunosuppression therapy. Nephrology. 2017;22:796–803.

24. Gu G, Huang Y, Wu C, Guo Z, Ma Y, Xia Q, et al. Differential Expression of Long Noncoding RNAs During Cardiac Allograft Rejection. Transplantation. 2017;101:83–91.

25. Iyer MK, Niknafs YS, Malik R, Singhal U, Sahu A, Hosono Y, et al. The landscape of long noncoding RNAs in the human transcriptome. Nat Genet. 2015;47:199–208.

26. Han Y, Eppinger E, Schuster IG, Weigand LU, Liang X, Kremmer E, et al. Formin-like 1 (FMNL1) Is Regulated by N-terminal Myristoylation and Induces Polarized Membrane Blebbing. J Biol Chem. 2009;284:33409–17.

27. Sun Z, Jing C, Xiao C, Li T. Long Non-Coding RNA Profile Study Identifies an Immune-Related lncRNA Prognostic Signature for Kidney Renal Clear Cell Carcinoma. Front Oncol. 2020;10:1430.

28. Zhao J, Lin X, Zhuang J, He F. Relationships of N6-Methyladenosine-Related Long Non-Coding RNAs With Tumor Immune Microenvironment and Clinical Prognosis in Lung Adenocarcinoma. Front Genet. 2021;12:714697.

29. Xu M, Chen Y, Lu W, Kong L, Fang J, Li Z, et al. SPMLMI: predicting lncRNA–miRNA interactions in humans using a structural perturbation method. PeerJ. 2021;9:e11426.

30. Yoon J-H, Abdelmohsen K, Gorospe M. Functional interactions among microRNAs and long noncoding RNAs. Semin Cell Dev Biol. 2014;34:9–14.

31. Ragusa M, Barbagallo C, Brex D, Caponnetto A, Cirnigliaro M, Battaglia R, et al. Molecular Crosstalking among Noncoding RNAs: A New Network Layer of Genome Regulation in Cancer. Int J Genomics. 2017;2017:1–17.

32. Wu X, Ding M, Liu Y, Xia X, Xu F, Yao J, et al. hsa-miR-3177-5p and hsa-miR-3178 Inhibit 5-HT1A Expression by Binding the 3′-UTR Region in vitro. Front Mol Neurosci. 2019;12:13.

33. Shen W, Huang B, He Y, Shi L, Yang J. Long non‐coding RNA RP11‐820 promotes extracellular matrix production via regulating miR‐3178/MYOD1 in human trabecular meshwork cells. FEBS J. 2020;287:978–90.

34. Wang H, Li K, Mei Y, Huang X, Li Z, Yang Q, et al. Sp1 Suppresses miR-3178 to Promote the Metastasis Invasion Cascade via Upregulation of TRIOBP. Mol Ther Nucleic Acids. 2018;12:1–11.

35. Wu J, Liu Z, Ding X, Ke R. miR-3178 as a prognostic indicator and tumor suppressor of gastric cancer. Irish Journal of Medical Science (1971 -). 2022;191:139–45.

36. Gu J, Huang W, Wang X, Zhang J, Tao T, Zheng Y, et al. Hsa-miR-3178/RhoB/PI3K/Akt, a novel signaling pathway regulates ABC transporters to reverse gemcitabine resistance in pancreatic cancer. Mol Cancer. 2022;21:112.

37. Wheeler A, Ridley A. Why three Rho proteins? RhoA, RhoB, RhoC, and cell motility. Exp Cell Res. 2004;301:43–9.

38. Skelton TS, Tejpal N, Gong Y, Kubiak JZ, Kloc M, Ghobrial RM. Allochimeric molecules and mechanisms in abrogation of cardiac allograft rejection. J Heart Lung Transplant. 2012;31:73–84.

39. Wu J, Zhang H, Zheng Y, Jin X, Liu M, Li S, et al. The Long Noncoding RNA MALAT1 Induces Tolerogenic Dendritic Cells and Regulatory T Cells via miR155/Dendritic Cell-Specific Intercellular Adhesion Molecule-3 Grabbing Nonintegrin/IL10 Axis. Front Immunol. 2018;9:1847.

40. Zhang M, Zheng Y, Sun Y, Li S, Chen L, Jin X, et al. Knockdown of NEAT1 induces tolerogenic phenotype in dendritic cells by inhibiting activation of NLRP3 inflammasome. Theranostics. 2019;9:3425–42.

41. Tubita V, Segui-Barber J, Lozano JJ, Banon-Maneus E, Rovira J, Cucchiari D, et al. Effect of immunosuppression in miRNAs from extracellular vesicles of colorectal cancer and their influence on the pre-metastatic niche. Sci Rep. 2019;9:11177.
